# HIV-AIDS prevention in the conception of HIV-positive young people

**DOI:** 10.11606/s1518-8787.2019053001174

**Published:** 2019-09-18

**Authors:** Stella Regina Taquette, Luciana Maria Borges da Matta Souza

**Affiliations:** I Universidade do Estado do Rio de Janeiro. Faculdade de Ciências Médicas. Departamento de Pediatria. Rio de Janeiro, RJ, Brasil; II Universidade Estácio de Sá. Faculdade de Medicina. Rio de Janeiro, RJ, Brasil

**Keywords:** Young Adult, psychology, Acquired Immunodeficiency Syndrome, prevention & control, HIV Infections, prevention & control, Health Knowledge, Attitudes, Practice, Qualitative Research

## Abstract

**OBJECTIVE:**

To analyze the conception of seropositive young people on how to prevent HIV infection.

**METHODS:**

This is a qualitative study using semi-structured interviews with HIV-positive young people whose diagnosis was made in adolescence 5 years ago or less. We followed a semi-structured script containing sociodemographic data and an open question on HIV/AIDS prevention. The interviews were recorded and fully transcribed, then analyzed with the support of the webQDA software. We used the categories that compose the concept of vulnerability as a theoretical basis for data analysis.

**RESULTS:**

We interviewed 39 young people, 23 girls and 16 boys. Some perceive the prevention of HIV infection only as an individual issue, summarizing it to the use of condoms and self-care. Most of the interlocutors point out educational strategies as the most relevant for prevention but used in a permanent and non-punctual way. In schools, they believe it is necessary to include younger students and their family. Guidelines should be given by people who can use the language of young people and preferably by HIV-positive people, to show the reality of those who have AIDS. In the programmatic field, they suggest intensifying campaigns in the media, distributing condoms in large scale, producing vaccines and medicines that cure. No one mentioned the female condom, the rapid test, nor the availability of sexual and reproductive health care.

**CONCLUSIONS:**

The qualification and expansion of communication strategies on sexuality in schools is urgent and essential in HIV and AIDS prevention in adolescence, contrary to the current trend of restricting the discussion of these topics in education policies.

## INTRODUCTION

According to the Epidemiological Report on HIV/AIDS of the Brazilian Ministry of Health, referring to cases reported up to June 2017^1^ , the population group aged 15 to 19 continues to show increasing rates of AIDS incidence. In the distribution by sex, the detection rate among women in the last 10 years decreased in all age groups, except between 15 and 19 years. The prevalence ratio between sexes, of 17 men to every 10 women, is lower in this age group when compared with the others, showing that the reduction in the trend of feminization of the epidemy observed from 2009 was lower in adolescents. In the 20-29 age group, this ratio is 30 men to every 10 women. In the case of men, the detection rate tripled between 15 and 19 years in the same period, from 2.4 to 6.9 cases per 100 thousand inhabitants.

A recent national survey of HIV surveillance in a sample of 4,176 men who have sex with men (MSM), performed in 12 municipalities in the five macro regions of Brazil, estimated HIV prevalence of 18.4%, which is significantly higher than the prevalence of 12.1% found in 2009 in a similar study. Regarding the age group, the study showed a higher prevalence increase between 15 and 19 years, which tripled^2^ . AIDS in Brazil is out of control and goes against the world scenario^3^ .

Sexually transmitted infections (STIs) and AIDS in adolescents are serious problems^4^ and are still almost invisible in the field of health. The Brazilian population in the age group from 10 to 19 years is expressive, about 17.9% of the total; when added to the young population, up to 24 years, it corresponds to 26.7% of the total of Brazilians^5^ . This population contingency is, undoubtedly, one of society’s great challenges for a better future. It is noteworthy that, although AIDS is the second leading cause of death among adolescents worldwide, its overall impact is often invisible^6^ .

In view of the observed increase in HIV incidence rates, new prevention strategies are needed. The objective of this study was to analyze the conception/perception of HIV positive adolescents on how to prevent HIV infection among adolescents in order to provide subsidies to public policies.

## METHODS

This study is an excerpt of a larger research on AIDS in adolescents in the city of Rio de Janeiro developed in 2011^7 , 8^ . The target audience was composed of adolescents/young people under treatment. Given the nature of the object in question, the method chosen was qualitative, with a semi-structured interview as a technique for collecting information. The interview allows us to know, through the narratives of the interlocutors, the value system of the investigated social group and reveals its specific structural, socioeconomic and cultural conditions.

To compose a varied sample of subjects, we recruited possible participants of the research in four large general hospitals of the city of Rio de Janeiro: Hospital Universitário Pedro Ernesto, Hospital Universitário Gaffrée e Guinle, Hospital Universitário Clementino Fraga Filho, and Hospital Federal dos Servidores do Estado. These establishments provide care to patients coming from different neighborhoods and from different social classes, predominantly with a lower socioeconomic level.

The services were previously contacted by the team of researchers, consisting of a physician, a social worker and a nurse, who requested permission to conduct the research, as well as collaboration in the referral of the patients to be interviewed. Data collection started after the consent of participants and, when they were under 18 years-old, of their guardians.

The study included only patients who were diagnosed with AIDS between 10 and 19 years of age and with illness time of up to 5 years, in order to ensure a certain homogeneity in the group regarding the duration of the disease. The staff attended hospitals at least twice a week for 18 months. When the service professionals met young people who met the inclusion criteria, they were referred to the interviewers. None of the patients referred refused to participate. We closed data collection when saturation of the collected information occurred.

The interviews were based on a previously screened questionnaire containing questions about demographic, family and sexual data, history of infection/diagnosis of the disease, and, finally, an open question on how to prevent HIV infection in adolescents. All the meetings were recorded and fully transcribed. From the beginning and during the study, the textual data derived from the transcriptions were analyzed according to hermeneutic-dialectic principles, as outlined by Minayo^9^ , and supported by the webQDA software for qualitative data analysis^10^ . After reading and re-reading the texts, we identified the most relevant content by observing the similarities, divergences and contradictions in the narratives. Data were coded by the webQDA after identifying what was common in the narratives. We used as theoretical basis of analysis the categories that compose the concept of vulnerability, in which the chance of a person falling ill depends on a set of individual, social and programmatic aspects^11^ . We sought to identify the meanings attributed by the subjects to the raised question, in order to understand the internal logic of this group, in a comparative dialogue with literature. At the end, an interpretative synthesis was elaborated to answer the questioning of the study.

The research complies with the ethical principles contained in the Declaration of Helsinki and was approved by the Research Ethics Committee of the Municipal Health Department (Opinion 262A). All interviewees and, when they were under 18 years-old, their guardians, signed the Informed Consent Form. No previous contact was established between the researchers and study participants.

## RESULTS

We conducted 39 interviews, being 16 boys and 23 girls. Most (82%) belonged to the lower socioeconomic class with family incomes of less than or equal to five minimum wages. As for schooling, more than half of the participants had a school delay greater than two years. Self-reported race/color in 66.6% of them was black (50% black and 50% brown). In relation to the route of exposure to HIV, in both sexes, the most frequent was sexual. For women, this route of exposure was heterosexual, except for two cases (one through blood and one unknown); for men, 75% was homosexual and 25% heterosexual.

In the analyses already carried out in the larger study, both sexes did not believe in the possibility of contamination as a situation of vulnerability to HIV. Respondents did not believe they could become infected, despite the absence of self-care and safety in sexual relations. For the infected boys, other prominent occurrences were sexual subjection, homophobia and commercial sexual exploitation. More than 90% of the boys did not have any emotional ties with those who infected them. In addition, 81.3% of them had more than four sexual partnerships and 37.5% had prostituted themselves. For young women, the vulnerability contexts that stand out are situations of violence, including the low age of sexual initiation (in 56.5% of cases, the first sexual relation occurred between 10 and 14 years), and HIV infection by their own partners (69.6% of them had an emotional bond with the contaminant)^7 , 8^ .

The narratives of the interviewees on HIV/AIDS prevention were classified into three theoretical categories, which compose the concept of vulnerability^9^ : individual actions, social actions and programmatic actions.

### Individual Actions

When talking about prevention, some have shown some skepticism when stating that there is nothing to be done to prevent it, as it depends on the behavior of each. No lack of knowledge was observed, and every young person knows how to avoid HIV infection. If you do not prevent it, it is for lack of self-care. They blame the adolescent him/herself for the risk of becoming infected. They criticize those who do not think about the consequences of their acts and also those who, by consuming alcoholic beverages and drugs, do not remember to use a condom during sex. For example:

*Nothing works. They tell us to wear a condom, but teenagers have sex without a condom.* (I14 – male)

*Those who want to protect themselves. The person who doesn’t wear a condom is stupid.* (I7 – female)

*I think the young mind is very twisted. And I think it’s no good at all, you know? Because most, not all, do not respect what others say. So, for them, whatever, it doesn’t matter.* (I26 – male)

*I think sometimes people get infected not because of a lack of information, no. Today, and I mean today, information is coming from everywhere. There are newspapers, campaigns, television, billboards, there’s a lot of information.* (I16 – male)

Other respondents suggested preventive measures related to self-care, such as use of condoms, awareness of what they are doing, and, for those who are infected, being careful not to contaminate their partners. They emphasized that, since sex is a very pleasant and often unexpected activity, young people should be careful to always carry a condom with them.

### Social Actions

We classify in this category the narratives related to the relationship with the family, the media and sexual education strategies.

Most interviewed young people emphasized the need for qualification and expansion of educational actions, such as lectures and preventive campaigns. Others showed conservative thoughts, criticizing the early erotization of society and television media, which exposes images that would encourage people to have sex. They also censure the distribution of condoms.

*…that machine that gives condoms to young people? That’s ridiculous, they’re inducing the youth to have sex ahead of time.* (I13 – male)

The absence of family dialogue on issues related to sexuality was pointed out as a barrier to prevention. Our interlocutors say that the lack of dialogue and understanding about the subject in the family leads them to have difficulty talking about their doubts, fears and questions. Therefore, they suggest the inclusion of family members in prevention proposals. Educational work including parental involvement would foster rapprochement with children and facilitate dialogue.

*My mother and father have never talked to me about anything, and sex life begins very early. There’s no use, nowadays it’s not like it used to be.* (I17 – female)

*[There should] be a school education project that family members could access with their children.* (I21 – female)

*Schools should have some kind of project that would influence parents to talk more to their children about it.* (I1 – female)

Sexual orientation and HIV/AIDS prevention in schools should be intensified, according to the interviewees. In addition, they should be permanent and not punctual, focusing on the doubts of adolescents and providing early in adolescence, before the first sexual experience, an opportunity to discuss what they feel. There has been criticism of what has been done, which is considered insufficient to address the concerns of young people about sex.

*The ideal thing would be to put it into children’s heads still in school.* (I1 – female)

*I think there’s little information, very little information really… It needs to be more approached in schools, many times… It has to be extremely repetitive until it becomes a habit, a total consciousness.* (I32 – male)

*[They] have to talk more, talk always. Even more so now that the young people, like, I know boys and girls who live close to me that are 12 years-old and drink and smoke. These children don’t even know how to get a disease, you know?* (I39 – female)

*[This subject should be] discussed more openly in schools.* (I23 – male)

Other aspects strongly emphasized by the interviewees refer to the form and content of what is informed to young people. The guidelines should be offered in the language of adolescents, so as to facilitate their understanding, and preferably by young people and/or those who experience the infection. As for the content, it should be clear and straightforward, communicating without subterfuge or masks how is the life of someone who has AIDS, especially the difficulties they have to face. The content should stir up reflections on teenagers and even feelings of fear of the disease. This fear would be positive in the opinion of some, as it would cause them to be more careful about their own health.

*[They should] speak in the language of the adolescent. Instead of using a 30-year-old, use an 18-year-old person, a 15, 16-year-old person.* (I23 - male)

*A language with lots of slangs… At some point [the young person] thinks, oh, this interests me, I want to know, understand?… If you talk like that, you can have a relationship with your girl, it’ll draw attention, because that is their language. If they don’t speak this language, they won’t understand… It’s no good, it’s going to continue in the same way.* (I23 – male)

The image of AIDS today is not the same as the beginning of the epidemic, according to the interviewees. The current youth do not know the disease as it was seen, practically a death sentence, so they find it necessary to have people living with AIDS to talk about the disease and the difficulties they go through.

*I have younger friends who say, “Oh, if I catch it, I’ll treat it myself.” Like it was so easy. It is a disease that has no cure yet. I live, but I wouldn’t want to have that kind of control in my life, I wouldn’t want to be in the hospital every month.* (I17 – female)

*You have to scare young people, showing them the injuries of STDs. A fear that does good. This fear is good, it’s nice. Because it makes you think before you do something. It might be a bit traumatic. But it’s something that the person will understand better in the future.* (I17 – female)

### Programmatic Actions

The actions suggested by the interviewees include the availability of preventive inputs, the inclusion of health professionals in schools to serve adolescents and provide preventive information, the production of vaccines and medicines that cure the disease, and the permanent investment in campaigns, and not just occasionally, as in Carnaval. None of the interviewees pointed out the need to offer health services aimed at adolescents, not even medical care for sexual and reproductive demands. Nor was the rapid test for diagnosis of seropositivity pointed out by any of the interlocutors.

Respondents emphasized, as a government strategy, the need to distribute condoms on a large scale, in a debureaucratized and confidential way, without identification of who is getting them. The girls suggested that the condom dispensing machines should be located in school lavatories, so they would not be seen or slandered for taking a condom. There was no mention of the use of a female condom by any of the interlocutors.

*You need to have a condom.* (I25 – female)

*…more condoms should be distributed. But to get a condom, the health center needs [identification].* (I3 – male)

*They put it in a very public area. If the girl gets it, she’s a whore. If the boy gets it, he’s a Don Juan.* (I17 – female)

The presence of a health professional in schools was considered important, since it could offer more knowledge and clear up the doubts of young people. After all, it is at school that teenagers learn most. The information should be given clearly and without subterfuge, including in the campaigns, with greater dissemination in the media. They emphasized the importance of investing in the production of vaccines and more effective medicines.

*There should be a health professional at school, who shows pictures of how the person [with AIDS] looks.* (I22 – male)

*The government only talks about condoms at Carnaval. The whole year people are getting sick, so it has to be advertised all year long. [They should] distribute the resources they spend at Carnaval all year round.* (I27 – male)

*The lectures are scarce, nurses or doctors should give lectures on this, distribute more condoms.* (I3 – male)

*[They should] make a vaccine or something to kill it. So far none of this has happened.* (I8 – male)

*I think it has to involve the media, a better explanation, since it’s still a very little talked about subject.* (I20 – female)

## DISCUSSION

The prevention proposals referred to by the young people interviewed are not original. In decades of AIDS epidemic, we have noticed that behavioral pattern changes did not happen deep enough to change the course of the disease. This shows that the efforts undertaken by public policies have not been able to account for significant changes in the cultural pattern of response to the epidemic. However, the narratives of the interlocutors point to strategies that contribute to the effectiveness of communication. For some, prevention depends exclusively on the individual, without perceiving the context as a vulnerability factor^12^ . The fact that young people, in the hour of pleasure, do not think about the consequences of unprotected sex reinforces that the question of sexual excitement cannot be underestimated when one thinks of prevention, nor other psychosocial barriers that make the use of condoms difficult, such as the consumption of alcohol and drugs^13 , 14^ .

Family participation was repeatedly mentioned as important in prevention activities. Educating the family contributes to the expansion of the dialogue between parents and adolescent children and to the extension of the technical knowledge on the subject. Despite the parents’ interest in talking to their children, they do not feel prepared and often do so superficially^15 , 16^ . Expanding parental knowledge and standardizing concepts and language is of great relevance, as adolescents sometimes receive information at school that goes against family behaviors, such as the distribution of condoms in schools^17^ .

The school appeared in the statements of the interviewees as the main scenario for HIV/AIDS prevention activities. However, the way they have been accomplished is not satisfactory and does not achieve its goals. The language of the activities should change, the frequency in which they are taught as well as their content need to be expanded. Knowledge about the disease is fundamental, since the perception of its severity is one of the most important factors associated with susceptibility to HIV-AIDS^18 , 19^ .

In Brazil, the first experiments with sex education in the school were performed in the 1960s^20^ and had a hygienist character. From 1970, with the demands of the feminist movement, this situation began to change, but it was only with the end of dictatorship and political opening in the 1990s that more effective educational proposals emerged, but still with a biological bias^21^ . In 1995, the government included sexuality in the National Curricular Parameters as a cross-cutting theme, articulated to others, such as ethics, health, gender, ecology and cultural plurality. It should be worked in a continuous, systematic way, integrated to the educational work in the school. Within this perspective, the *Programa Saúde e Prevenção na Escola* (SPE – Health and Prevention at School Program)^22^ was created in 2006, a partnership between the Ministry of Education and the Ministry of Health aimed at reaching the specific target audience of school adolescents, with its main actions focused on sexual and reproductive health of adolescents and young people. This government policy, however, was replaced in 2010 by the *Programa Saúde na Escola* (PSE – Health in School Program), which implemented its actions in a larger context, included other age groups, and did not prioritize issues related to sexuality^23^ . All of these changes over the years in relation to the policies developed in the area of education aimed at adolescents promoted an expansion of school coverage, according to a study by Neves and Romero^24^ ; however, the actions had low effectiveness.

Currently, in addition to the low effectiveness of policies in this field, movements contrary to it are evident, such as the withdrawal of the terms gender and sexual orientation from the text of the National Education Plan, approved in 2014, provoked by the religious bench of the Brazilian National Congress. Another movement worthy of note is the No-Party School – Bill No.193/2016^25^ , which poses a serious threat to an emancipatory and rights-based education, whose ideology, contrary to when it says that it is aimed at neutrality, seeks to prevent debates and pedagogical practices related, among others, to questions about gender and sexuality. In the field of prevention, Brazil is retreating^26^ .

International studies also emphasize the need for more effective educational strategies as a result of insufficient knowledge on HIV transmission^27^ . In France, for example, sexual education in school is a legal requirement and HIV prevalence rates are approximately three times lower than those in Brazil^28 , 29^ . Another French standard is the school health service, which has permanent nursing professionals in all secondary schools^30^ .

None of the interviewees included health services as necessary for the prevention of HIV/AIDS, and the difficulties of access to services are one of the factors that keep these social groups in more vulnerable conditions. In the city of Rio de Janeiro, a study on sexual and reproductive health services for adolescents showed that only 12.9% of the units perform educational activities and less than 1/3 of the physicians are trained to attend this public^31^ . It is worth mentioning that it is the responsibility of the *Estratégia Saúde da Família* (ESF – Family Health Strategy) teams to identify adolescents in a situation of social and personal vulnerability in the territory, to intervene to improve quality of life, and to promote support actions, social inclusion, protection and guarantee of rights, including school spaces^32^ . Paiva et al.^33^ indicate that no action is successful without considering the sociopolitical context, including recognizing the importance of working with vulnerable populations and the need for special measures for each situation, as in the case of adolescents.

## FINAL CONSIDERATIONS

The study, although restricted to the perception of young people who have AIDS, brings relevant contributions, which can be included in HIV prevention programs in the adolescent age: the distribution of condoms in a debureaucratized, confidential and large scale manner; and more effective and permanent strategies for sexuality education in schools, including the family, starting early and taught in a language young people understand, showing what life is like for those with AIDS.

Currently, a greater emphasis on the use of pre- and post-exposure to HIV drugs is observed as a preventive public policy, which does not seem to be sufficient if, for example, we look at the use of such a preventive strategy against syphilis, without satisfactory results. Other preventive policies and services for sexual and reproductive health care should be used, especially for the most vulnerable groups, in addition to permanent government campaigns.

It is important to highlight the limits of this study, since the research in which it was based only the vision of a very specific portion of the population stratum of adolescents, that is, those already infected. Another limitation of the study is its territorial coverage, reduced to one large municipality. However, we believe this study provides support for coping more effectively with the HIV/AIDS epidemic in the investigated population stratum.
