# Supplementary material for: HIV-AIDS prevention in the conception of HIV-positive young people
Source: Rev Saude Publica. 2019 Sep 18;53:80. doi: 10.11606/s1518-8787.2019053001174 (PMC6763283; doi:10.11606/s1518-8787.2019053001174)
Supplement: Translation

# Translation: Prevenção de HIV-Aids na concepção de jovens soropositivos

**Authors:** Stella Regina Taquette, Luciana Maria Borges da Matta Souza

**Affiliations:** I Universidade do Estado do Rio de Janeiro. Faculdade de Ciências Médicas. Departamento de Pediatria. Rio de Janeiro, RJ, Brasil; II Universidade Estácio de Sá. Faculdade de Medicina. Rio de Janeiro, RJ, Brasil

**Keywords:** Adulto Jovem, psicologia, Síndrome de Imunodeficiência Adquirida, prevenção & controle, Infecções por HIV, prevenção & controle, Conhecimentos, Atitudes e Prática em Saúde, Pesquisa Qualitativa

## Abstract

**OBJETIVO:**

Analisar a concepção de jovens soropositivos sobre como prevenir a infecção pelo HIV.

**MÉTODOS:**

Estudo qualitativo por meio de entrevistas semiestruturadas com jovens soropositivos cujo diagnóstico foi feito na adolescência havia no máximo 5 anos. Seguimos roteiro semiestruturado contendo dados sociodemográficos e pergunta aberta sobre prevenção de HIV-Aids. As entrevistas foram gravadas e transcritas na íntegra, sendo analisadas com apoio do software webQDA. Utilizamos as categorias que compõem o conceito de vulnerabilidade como base teórica para a análise dos dados.

**RESULTADOS:**

Entrevistamos 39 jovens, 23 do sexo feminino e 16 do masculino. Alguns percebem a prevenção da infecção pelo HIV apenas como uma questão individual, resumindo-a ao uso do preservativo e ao autocuidado. A maior parte dos interlocutores aponta estratégias educativas como as mais relevantes para a prevenção, mas utilizadas de forma permanente e não pontual. Nas escolas, acreditam ser necessário incluir alunos mais novos e a família. As orientações devem ser ministradas por pessoas que utilizam a linguagem dos jovens e de preferência por soropositivos, para mostrar a realidade da vida de quem tem Aids. No campo programático, indicam intensificação das campanhas na mídia, distribuição de camisinha em larga escala, produção de vacinas e medicamentos que curem. Não houve menção ao preservativo feminino, ao teste rápido, à disponibilidade de atendimento em saúde sexual e reprodutiva.

**CONCLUSÕES:**

A qualificação e ampliação das estratégias de comunicação sobre sexualidade nas escolas é urgente e essencial na prevenção de HIV e Aids na adolescência, ao contrário da tendência que hoje se verifica de restrição da discussão sobre esses temas nas políticas de educação.

## INTRODUÇÃO

De acordo com o Boletim Epidemiológico sobre HIV/Aids do Ministério da Saúde do Brasil, referente aos casos notificados até junho de 2017^1^ , o grupo populacional entre 15 e 19 anos continua apresentando taxas crescentes de incidência de Aids. Na distribuição por sexo, verifica-se uma redução da taxa de detecção entre as mulheres nos últimos 10 anos em todas as faixas etárias, exceto entre 15 e 19 anos. A razão de prevalência entre os sexos, de 17 homens para cada 10 mulheres, é menor nessa faixa etária quando comparada às demais idades, evidenciando que a redução da tendência de feminização da epidemia observada a partir de 2009 foi menor em adolescentes. Na faixa etária de 20 a 29 anos, essa razão é de 30 homens para cada 10 mulheres. No caso dos homens, a taxa de detecção triplicou entre 15 e 19 anos no mesmo período, de 2,4 para 6,9 casos por 100 mil habitantes.

Pesquisa nacional recente de vigilância do HIV em uma amostra de 4.176 homens que fazem sexo com homens (HSH), realizada em doze municípios das cinco macrorregiões do Brasil, estimou prevalência de 18,4% de HIV, significativamente maior do que a prevalência de 12,1% encontrada em 2009, em estudo semelhante. Em relação à faixa etária, o estudo mostrou maior aumento da prevalência entre 15 e 19 anos, que triplicou^2^ . A Aids no Brasil mostra-se em descontrole e está na contramão do cenário mundial^3^ .

As infecções sexualmente transmissíveis (IST) e a Aids em adolescentes são problemas graves^4^ e ainda pouco visíveis no campo da saúde. A população brasileira na faixa etária de 10 a 19 anos é expressiva, cerca de 17,9% do total; quando somada à população jovem, de até 24 anos, corresponde a 26,7% do total de brasileiros^5^ . Esse contingente populacional representa, sem dúvida, um dos grandes desafios da sociedade para que um futuro melhor lhe seja garantido. Importante destacar que, apesar de a Aids ser a segunda causa de morte entre adolescentes no mundo, seu impacto global é frequentemente invisível^6^ .

Diante do aumento observado das taxas de incidência de HIV, são necessárias novas estratégias de prevenção. Este estudo teve por objetivo analisar a concepção/percepção de jovens soropositivos sobre como prevenir a infecção pelo HIV entre adolescentes com vistas a oferecer subsídios às políticas públicas.

## MÉTODO

Este estudo é um recorte de pesquisa maior sobre Aids em adolescentes no município do Rio de Janeiro desenvolvida em 2011^7 , 8^ . O público-alvo foi composto por adolescentes/jovens em tratamento. Dada a natureza do objeto em questão, o método escolhido foi qualitativo e a entrevista, semiestruturada como técnica de coleta das informações. A entrevista nos permite conhecer por meio das narrativas dos interlocutores o sistema de valores do grupo social investigado e é reveladora de suas condições estruturais, socioeconômicas e culturais específicas.

Para compor uma amostra de sujeitos variada, recrutamos os possíveis participantes da pesquisa em quatro grandes hospitais gerais do município do Rio de Janeiro: Hospital Universitário Pedro Ernesto, Hospital Universitário Gaffrée e Guinle, Hospital Universitário Clementino Fraga Filho e Hospital Federal dos Servidores do Estado. Esses estabelecimentos prestam atendimento a pacientes advindos de diversos bairros e de variadas classes sociais, predominantemente de nível socioeconômico inferior.

Os serviços foram contatados previamente pela equipe de pesquisadores, composta por médica, assistente social e enfermeira, que solicitaram permissão para a realização da pesquisa, bem como colaboração no encaminhamento dos pacientes a serem entrevistados. Após a anuência dos participantes e de seus responsáveis, quando menores de 18 anos, foi iniciada a coleta de dados.

Foram incluídos na pesquisa somente pacientes com diagnóstico de Aids realizado entre 10 e 19 anos e com tempo de adoecimento de até 5 anos, para garantir certa homogeneidade no grupo quanto à duração da doença. A equipe frequentou os hospitais no mínimo duas vezes por semana durante 18 meses. Quando os profissionais dos serviços atendiam jovens que preenchiam os critérios de inclusão, eles eram encaminhados às entrevistadoras. Não houve recusas dos pacientes encaminhados. Encerramos a coleta de dados quando avaliamos ter ocorrido saturação das informações colhidas.

As entrevistas realizadas obedeceram a roteiro previamente testado, contendo questões sobre dados demográficos, familiares, histórico sexual e da infecção/diagnóstico da doença e, ao final, uma pergunta aberta sobre como prevenir a infecção de HIV em adolescentes. Todos os encontros foram gravados e transcritos na íntegra. Desde o início e no decorrer do estudo, procedeu-se à análise dos dados textuais oriundos das transcrições de acordo com princípios hermenêutico-dialéticos balizados por Minayo^9^ e com o apoio do software webQDA de análise qualitativa de dados^10^ . Após leitura e releitura dos textos, identificamos o conteúdo mais relevante por meio da observação das semelhanças, divergências e contradições nas narrativas. Os dados foram codificados pelo webQDA após identificação do que havia de comum nas narrativas. Utilizamos como base teórica de análise as categorias que compõem o conceito de vulnerabilidade, no qual a chance de a pessoa adoecer depende de um conjunto de aspectos individuais, sociais e programáticos^11^ . Buscamos identificar os sentidos atribuídos pelos sujeitos à questão levantada, para entender a lógica interna desse grupo, em diálogo comparativo com a literatura. Ao final, elaborou-se uma síntese interpretativa com vistas a responder o questionamento do estudo.

A pesquisa cumpre os princípios éticos contidos na Declaração de Helsinki, tendo sido aprovada pelo Comitê de Ética em Pesquisa da Secretaria Municipal de Saúde (Parecer 262A). Todos os entrevistados e seus responsáveis, quando menores de 18 anos, assinaram o Termo de Consentimento Livre e Esclarecido. Não houve contato prévio entre os pesquisadores e os participantes do estudo.

## RESULTADOS

Realizamos 39 entrevistas, sendo 16 rapazes e 23 moças. A maioria deles (82%) pertencia às camadas sociais de menor poder econômico com renda familiar menor ou igual a cinco salários mínimos. Quanto à escolaridade, verificamos atraso escolar maior que dois anos em mais da metade dos participantes. A raça/cor autorreferida em 66,6% deles foi negra (50% pretos e 50% pardos). Em relação à via de exposição ao HIV, em ambos os sexos, a mais frequente foi a sexual. Para as mulheres, a heterossexual, exceto em dois casos (uma sanguínea e outra desconhecida); para os homens, 75% homossexual e 25% heterossexual.

Nas análises já realizadas no estudo maior, evidenciou-se, em ambos os sexos, a descrença na possibilidade de contaminação como situação de vulnerabilidade ao HIV. Os entrevistados não acreditavam que pudessem se infectar, apesar da ausência do autocuidado e de segurança nas relações sexuais. Para os rapazes infectados, outras ocorrências destacadas foram a sujeição sexual, a homofobia e a exploração sexual comercial. Mais de 90% dos rapazes não tinham qualquer vínculo afetivo com quem os infectou. Além disso, 81,3% deles tiveram mais de quatro parcerias sexuais e 37,5% se prostituíam. Para as moças, ressaltam-se como contextos de vulnerabilidade a vivência de situações de violência, incluindo a baixa idade da iniciação sexual (em 56,5% delas a sexarca ocorreu entre 10 e 14 anos), e o contágio de HIV pelos seus próprios parceiros (69,6% delas tinham vínculo afetivo com o contaminante)^7 , 8^ .

As narrativas dos entrevistados sobre a prevenção de HIV-Aids foram classificadas em três categorias teóricas, que compõem o conceito de vulnerabilidade^9^ : ações individuais, ações sociais e ações programáticas.

### Ações Individuais

Ao falar sobre prevenção, alguns demonstraram certo ceticismo ao afirmar que não há nada a ser feito para prevenir, pois depende do comportamento de cada um. Não há falta de conhecimento, e todo jovem sabe como evitar o contágio pelo HIV. Se não faz prevenção, é por falta de autocuidado. Culpam o próprio adolescente pelo risco que corre de se infectar. Criticam os que não pensam nas consequências de seus atos e também aqueles que, por consumirem bebidas alcoólicas e drogas, na hora do sexo não lembram de usar o preservativo. Vejamos alguns exemplos:

*Nada adianta. Eles falam para usar camisinha, mas os adolescentes transam sem camisinha.* (E14 – sexo masculino)

*Não se previne quem não quer. Burra é a pessoa que não usa camisinha.* (E7 – sexo feminino)

*Eu acho que a mente jovem tá muito virada. E eu acho que não adianta nada, entendeu? Porque a maioria, não todos, não respeita o que os outros falam. Então pra eles tanto faz como tanto fez, ficar em cima ou não ficar.* (E26 – sexo masculino)

*Acho que às vezes as pessoas se contaminam não é por falta de informação, não. Hoje, eu digo é por hoje, a informação está chegando por tudo quanto é canto. Tem jornal, tem campanha, televisão, tem outdoor, tem um monte de coisa informando.* (E16 – sexo masculino)

Outra parte dos entrevistados sugeriu medidas preventivas relacionadas ao autocuidado, como o uso de preservativo, ter consciência do que está fazendo e, para aqueles que estão infectados, tomar cuidado para não contaminarem seus parceiros e parceiras. Ressaltaram que como o sexo é uma atividade muito prazerosa e, muitas vezes, inesperada, o jovem deveria se precaver carregando sempre consigo o preservativo.

### Ações Sociais

Classificamos nesta categoria as narrativas referentes ao relacionamento com a família, aos meios de comunicação e às estratégias de educação em sexualidade.

A maioria dos jovens entrevistados enfatizou a necessidade de qualificação e ampliação de ações educativas, como palestras e campanhas preventivas. Outros mostraram pensamentos conservadores, criticando a erotização precoce da sociedade e a mídia televisiva, que expõe imagens que incentivariam as pessoas a praticarem sexo. Censuram também a distribuição de preservativo.

*... aquela maquininha de distribuir camisinha pros jovens? Aquilo ali é coisa ridícula, estão induzindo a juventude a fazer sexo antes do tempo.* (E13 – sexo masculino)

A ausência de diálogo familiar sobre temas relativos à sexualidade foi apontada como um entrave à prevenção. Nossos interlocutores referem que a falta de diálogo e de entendimento sobre o tema na família os levam a ter dificuldade de conversar sobre suas dúvidas, medos e questionamentos. Diante disso, sugerem a inclusão dos familiares nas propostas de prevenção. Os trabalhos educativos incluindo a participação dos pais propiciaria uma aproximação com os filhos e facilitaria o diálogo.

*Minha mãe e meu pai nunca conversaram nada comigo e a vida sexual começa muito cedo. Não adianta, hoje em dia não é mais como antigamente.* (E17 – sexo feminino)

*Ter um projeto de educação nas escolas que os familiares pudessem ter acesso junto com os filhos.* (E21 – sexo feminino)

*Devia ter algum tipo de trabalho na escola que influenciasse os pais a conversarem mais com os filhos sobre isso.* (E1 – sexo feminino)

As ações de orientação em sexualidade e prevenção de HIV-Aids realizadas nas escolas deveriam ser intensificadas, segundo os entrevistados. Além disso, deveriam ser permanentes e não pontuais, focando nas dúvidas dos adolescentes e proporcionando logo no início da adolescência, antes da primeira experiência sexual, oportunidade para discutirem sobre o que sentem. Houve críticas ao que tem sido feito, sendo considerado insuficiente para atender às inquietações dos jovens sobre sexo.

*O mais interessante seria colocar na cabeça das crianças já na escola.* (E1 – sexo feminino)

*Eu acho que tem muito pouca informação, muito pouca informação mesmo... Tem que ser muito abordado nas escolas, muitas vezes... Tem que ser extremamente repetido até que se torne um hábito, uma consciência total.* (E32 – sexo masculino)

*Tem que falar mais, ficar falando sempre. Ainda mais agora que os jovens, tipo, tenho perto de casa meninos e meninas de 12 anos que bebem e fumam. Essas crianças nem sabem como pega uma doença, entendeu?* (E39 – sexo feminino)

*É falando mais abertamente nas escolas.* (E23 – sexo masculino)

Outros aspectos muito enfatizados pelos entrevistados se referem à forma e ao conteúdo do que é informado aos jovens. As orientações deveriam ser ofertadas na linguagem dos adolescentes, de maneira a lhes facilitar o entendimento e, de preferência, por pessoas jovens e/ou que vivam a experiência da infecção. Quanto ao conteúdo, deveria ser claro e direto, comunicando sem subterfúgios ou máscaras como é a vida de alguém que tem Aids, principalmente as dificuldades que tem de enfrentar. O conteúdo deveria provocar reflexões nos adolescentes e até sentimentos de medo da doença. Esse medo seria positivo na opinião de alguns, pois faria com que tivessem mais cuidado com a própria saúde.

*Falar na linguagem do adolescente. Em vez de botar uma pessoa de 30 anos, bota uma pessoa de 18, uma pessoa de 15, 16.* (E23 – sexo masculino)

*Uma linguagem com muita gíria... Em dado momento ele vai, opa, aquilo já me interessa, eu quero saber, entendeu? ... Você diz assim, você pode ter relação com sua mina, vai dar aquele ibope, porque essa é a linguagem dele. Se não falar essa linguagem, eles não vão entender... Não vai adiantar nada, vai continuar do mesmo jeito.* (E23 – sexo masculino)

A imagem da Aids de hoje não é a mesma do início da epidemia, segundo os entrevistados. A juventude atual não conhece a doença como era vista, praticamente uma sentença de morte, por isso acham necessário apresentar as pessoas que vivem com Aids para que falem sobre a doença e as dificuldades pelas quais passam.

*Eu tenho amigas mais novas que falam assim: “Ah, se eu pegar, eu vou me tratar”. Como se fosse tão fácil. É uma doença que não tem cura ainda. Eu vivo, mas eu não queria ter esse tipo de controle na minha vida, não queria estar todo mês no hospital.* (E17 – sexo feminino)

*Tem que passar medo nos jovens, mostrando as lesões das DST. Um medo que faz bem. Esse medo é bom, é legal. Porque faz pensar antes de fazer. Tudo bem que pode ser uma coisa meio traumática. Mas é uma coisa que futuramente a pessoa vai entender melhor.* (E17 – sexo feminino)

### Ações Programáticas

As ações sugeridas pelos entrevistados incluem a disponibilização de insumos de prevenção, a inclusão de profissionais de saúde nos estabelecimentos escolares para atender os adolescentes e oferecer informações preventivas, a produção de vacinas e de medicamentos que curem a doença, e o investimento permanente em campanhas, e não apenas ocasionalmente, como no Carnaval. Nenhum entrevistado apontou a necessidade de oferta de serviços de saúde direcionados a adolescentes, nem mesmo de atendimento médico para demandas sexuais e reprodutivas. Tampouco o teste rápido para diagnóstico de soropositividade foi apontado por qualquer dos interlocutores.

Os entrevistados enfatizaram como estratégia de governo a necessidade de distribuição de preservativos em larga escala, de forma desburocratizada e sigilosa, sem identificação de quem está pegando. As moças sugeriram que as máquinas de distribuição dos preservativos estivessem localizadas nos banheiros das escolas, pois assim não seriam vistas, nem difamadas por pegarem o preservativo. Não houve menção de uso do preservativo feminino por nenhum dos interlocutores.

*Tem que ter o preservativo.* (E25 – sexo feminino)

*...tem que distribuir mais camisinha. Mas, para ter camisinha, o posto cobra cartão.* (E3 – sexo masculino)

*Eles tão botando numa área muito pública. Se a garota pega, é puta. Se o garoto pega, é garanhão.* (E17 – sexo feminino)

A presença de um profissional de saúde nas escolas foi considerada importante, pois poderia ofertar mais conhecimento e tirar as dúvidas dos jovens. Afinal, é na escola que os adolescentes mais aprendem. As informações deveriam ser dadas de forma clara e sem subterfúgios, inclusive nas campanhas, com maior divulgação na mídia. Enfatizaram a importância do investimento na produção de vacinas e de medicamentos mais eficazes.

*Tem que ter um profissional de saúde na escola, que mostre fotos de como a pessoa fica.* (E22 – sexo masculino)

*O governo só fala de camisinha no Carnaval. O ano inteiro o pessoal se fode pegando doença, então tem que ter propaganda o ano inteiro. Distribuir os recursos que eles gastam no Carnaval no ano inteiro.* (E27 – sexo masculino)

*As palestras são poucas, o enfermeiro ou o médico deveriam dar palestras sobre isso, distribuir mais camisinha.* (E3 – sexo masculino)

*Fazerem vacina ou acharem alguma coisa pra matar. Até agora nada disso aconteceu.* (E8 – sexo masculino)

*Eu acho que tem que ter uma mídia, uma explicação melhor que ainda é um assunto muito pouco falado.* (E20 – sexo feminino)

## DISCUSSÃO

As propostas de prevenção referidas pelos jovens entrevistados não são originais. Em décadas de epidemia da Aids, observamos que mudanças de padrão de comportamento não aconteceram de forma suficientemente profunda para alterar o percurso da doença. Isso evidencia que os esforços empreendidos pelas políticas públicas não foram capazes de dar conta de promover transformações significativas no padrão cultural de resposta à epidemia. Entretanto, as narrativas dos interlocutores apontam para estratégias que contribuem para a eficácia da comunicação. Para alguns, a prevenção depende exclusivamente do indivíduo, sem perceber o contexto como fator de vulnerabilidade^12^ . O fato de ressaltarem que o jovem, na hora do prazer, não pensa nas consequências do sexo sem proteção reforça que a questão da excitação sexual não pode ser menosprezada ao se pensar em prevenção, tampouco outras barreiras psicossociais que dificultam o uso do preservativo, como o consumo de álcool e drogas^13 , 14^ .

A participação da família foi reiteradamente citada como importante nas atividades de prevenção. Educar a família contribui para o alargamento do diálogo entre pais e filhos adolescentes e para a ampliação do conhecimento técnico sobre o tema. Apesar do interesse dos pais em conversar com os filhos, eles não se sentem preparados e, muitas vezes, o fazem de forma superficial^15 , 16^ . Expandir o conhecimento dos pais e uniformizar os conceitos e a linguagem é de grande relevância, pois, por vezes, os adolescentes recebem informações na escola que vão de encontro às condutas familiares, como a distribuição de preservativos nas escolas^17^ .

A escola apresentou-se nas falas dos entrevistados como o principal cenário para as atividades de prevenção de HIV-Aids. Entretanto, a forma como têm sido realizadas não é satisfatória e não alcança seus objetivos. A linguagem das atividades deve mudar, a frequência em que são ministradas bem como seu conteúdo precisam ser ampliados. Ter conhecimento sobre a doença é fundamental, pois a percepção de sua gravidade é um dos mais importantes fatores associados à susceptibilidade ao HIV-Aids^18 , 19^ .

No Brasil, as primeiras experiências com educação sexual na escola foram realizadas na década de 1960^20^ e tinham caráter higienista. A partir de 1970, com as demandas do movimento feminista, essa situação começou a se modificar, mas somente com o fim da ditadura e a abertura política nos anos de 1990 surgiram propostas educacionais mais efetivas, mas ainda com viés biologizante^21^ . Em 1995, o governo incluiu a sexualidade nos Parâmetros Curriculares Nacionais como tema transversal, articulado a outros, como ética, saúde, gênero, ecologia e pluralidade cultural. Deveria ser trabalhado de forma contínua, sistemática e integrada ao trabalho educativo na escola. Dentro dessa perspectiva, foi criado em 2006 o Programa Saúde e Prevenção na Escola (SPE)^22^ , uma parceria do Ministério da Educação com o Ministério da Saúde com vistas a atingir o público específico de adolescentes escolares, sendo suas ações principais voltadas à promoção da saúde sexual e reprodutiva de adolescentes e jovens. Essa política governamental, porém, foi substituída em 2010 pelo Programa Saúde na Escola (PSE), que encampou suas ações dentro de um contexto maior, incluiu outras faixas etárias e não priorizou as questões relacionadas à sexualidade^23^ . Todas essas mudanças ocorridas no decorrer dos anos em relação às políticas desenvolvidas na área da educação dirigida a adolescentes promoveram uma ampliação da cobertura das escolas, segundo estudo de Neves e Romero^24^ ; porém, as ações foram de baixa efetividade.

No momento atual, além da baixa efetividade das políticas nesse campo, evidencia-se movimentos contrários a ela, como a retirada dos termos gênero e orientação sexual do texto do Plano Nacional de Educação, aprovado em 2014, provocada pela bancada religiosa do Congresso Nacional brasileiro. Outro movimento digno de nota é o Escola Sem Partido – Projeto de Lei nº 193/2016^25^ , que representa uma grave ameaça a uma educação emancipadora e garantidora de direitos, cuja ideologia, ao contrário do que prega quando diz que visa à neutralidade, pretende impedir debates e práticas pedagógicas relacionadas, entre outras, a questões sobre gênero e sexualidade. No campo da prevenção, o Brasil está retrocedendo^26^ .

Estudos internacionais também enfatizam a necessidade de estratégias educativas mais eficazes em decorrência da insuficiência de conhecimento sobre transmissão de HIV^27^ . Na França, por exemplo, a educação sexual na escola é uma exigência legal e as taxas de prevalência de HIV são aproximadamente três vezes menores que as brasileiras^28 , 29^ . Outra normativa francesa é o serviço de saúde escolar, que conta com profissional da área de enfermagem permanente em todas os estabelecimentos de ensino de nível médio^30^ .

Nenhum dos entrevistados incluiu os serviços de saúde como necessários para a prevenção de HIV-Aids, e as dificuldades de acesso aos serviços é um dos fatores que mantêm esses grupos sociais em condições mais vulneráveis. No município do Rio de Janeiro, estudo sobre serviços de saúde sexual e reprodutiva para adolescentes evidenciou que apenas 12,9% das unidades realizam atividades educativas e menos de 1/3 dos médicos são capacitados para atender a esse público^31^ . Vale ressaltar que é atribuição das equipes da Estratégia Saúde da Família (ESF) identificar no território os adolescentes em situação de vulnerabilidade social e pessoal, intervir para favorecer a melhora da qualidade de vida e promover ações de apoio, inclusão social, proteção e garantia de direitos, incluindo os espaços escolares^32^ . Paiva et al.^33^ indicam que nenhuma ação é bem-sucedida sem considerar o contexto sociopolítico, incluindo o reconhecimento da importância do trabalho com populações vulneráveis e a necessidade de medidas especiais para cada situação, como no caso dos adolescentes.

## CONSIDERAÇÕES FINAIS

O estudo, apesar de restrito à percepção de jovens que têm Aids, traz contribuições relevantes, que podem ser incluídas nos programas de prevenção de HIV na faixa etária adolescente: a distribuição de preservativos de forma desburocratizada, confidencial e em larga escala; e estratégias de educação em sexualidade nas escolas mais eficazes e permanentes, incluindo a família, iniciando precocemente e ministradas em linguagem que o jovem entenda, mostrando como é a vida de quem tem Aids.

No momento atual, observa-se como política pública preventiva uma ênfase maior no uso de medicamentos pré e pós-exposição ao HIV, que não parece ser suficiente se olharmos, por exemplo, a utilização de semelhante estratégia preventiva no combate à sífilis, sem resultados satisfatórios. Outras políticas preventivas e serviços de atenção à saúde sexual e reprodutiva devem ser empregados, principalmente para os grupos de maior vulnerabilidade social, além de campanhas governamentais permanentes.

Cumpre ressaltar os limites deste trabalho, cuja pesquisa que lhe deu origem enfocou apenas a visão de uma parcela muito específica do estrato populacional de adolescentes, aqueles já infectados. Outra limitação do estudo é a sua abrangência territorial reduzida a um município de grande porte. No entanto, acreditamos que este estudo traz subsídios para o enfrentamento mais eficaz da epidemia de HIV-Aids no estrato populacional investigado.
